# Supplementary material for: Are carbon emissions trading and green financial instruments synergistic? -Comprehensive quantitative research based on content analysis
Source: PLoS One. 2024 Mar 7;19(3):e0298601. doi: 10.1371/journal.pone.0298601 (PMC10919601; doi:10.1371/journal.pone.0298601)
Supplement: S1 Data — (ZIP) [file pone.0298601.s001.zip › Data/地级市Z_F工作报告.zip!/║╙─╧/╜╣╫≈╩╨/└┤╫╘╣1⁄2╓┌║┼í╛┬φ┐╦╩2╛▌═°í┐_2014.docx]

各位代表：

现在，我代表市人民政府，向大会报告工作，请予审议，并请各位政协委员和列席人员提出意见。

一、过去五年工作回顾

市十一届人民代表大会第一次会议以来的五年，是我市改革发展进程中极不平凡的五年。在省委、省政府和市委的坚强领导下，市政府团结带领全市人民，深入贯彻落实科学发展观，抢抓中原经济区建设重大机遇，科学谋划、克难攻坚，全面加快经济转型示范市建设，经济社会发展迈上了新台阶，较好完成了市十一届人大确定的目标任务。

五年来，综合实力持续增强。2013年，全市地区生产总值达1707亿元，是2008年的1.7倍，年均增长11.7%，高于全省平均增速0.8个百分点；公共财政预算收入达97亿元，是2008年的1.9倍，年均增长14.6%。

五年来，发展方式持续转变。二、三产业比重提高0.6个百分点。高新技术产业增加值占规模以上工业增加值比重达23%，万元生产总值能耗下降23%。粮食总产“十连增”。AAAAA级景区3家，居全国地市级前列。

五年来，城乡建设持续推进。实施“14820”城市转型工程，中心城区建成区达108平方公里，城市组团、功能区和中心镇加快发展，社会主义新农村建设稳步推进。全市城镇化率达52%，提高6.7个百分点。

五年来，人民生活持续改善。城镇居民人均可支配收入、农民人均纯收入分别达22058元、11367元，是2008年的1.7倍、1.9倍，年均增长11%、13%。

主要做了以下工作：

（一）积极应对金融危机，保持经济平稳较快发展。面对国际金融危机严重冲击，努力扬长避短、危中寻机，牢牢把握经济工作主动权。坚持抓运行保态势。采取促进经济平稳较快增长50条措施，及时协调解决生产要素保障等问题。坚持上项目扩投资。实施省、市重点项目113个、1517个，完成投资403亿元、2314亿元。全社会固定资产投资年均增速达23%。坚持建载体强支撑。大力推进示范区和9个省级产业集聚区建设，2013年，示范区规模以上工业增加值、固定资产投资分别完成59亿元、96亿元，是组建时的3倍、4.5倍。9个省级产业集聚区建成面积达112平方公里，入驻规模以上企业453家；固定资产投资年均增长37%，3个产业集聚区进入全省“十强十快”行列。示范区和产业集聚区规模以上工业增加值占全市比重达60%以上。

（二）大力优化产业结构，加快转变经济发展方式。在全省率先谋划建设中原经济区经济转型示范市，并成功上升为省级战略，制订实施八大专项行动计划，强力推进经济转型示范市建设。加快新型工业化发展。大力实施工业强市，积极培育战略性新兴产业，改造提升传统支柱产业，形成了一批优势企业群体。2013年，规模以上工业增加值达987亿元，是2008年的1.8倍，年均增长15.6%。我市被列入全国资源型城市可持续发展规划，累计争取资源枯竭城市转移支付17亿元。强力实施节能减排，关停小火电机组150万千瓦，关闭污染企业104家，万元工业增加值能耗下降46%。加快现代农业发展。建成高标准粮田84万亩，发展节水灌溉面积43万亩。全省唯一的国家级台湾农民创业园落户我市。农民专业合作社3536家，农村土地流转面积96万亩。在全省率先实现主要粮食生产机械化。加快文化旅游业发展。云台山景区荣获国家生态旅游示范区称号、首届省长质量奖，神农山、青天河成功创建国家AAAAA级景区，圆融寺、嘉应观、影视城、陈家沟成功创建国家AAAA级景区。累计接待游客1亿人次、旅游综合收入近900亿元，连续4年荣获中国旅游竞争力百强城市称号。加快商贸物流业发展。扎实推进万村千乡市场工程、社区便民服务工程、家电下乡工程，大力发展物流业，积极推进商务中心区和特色商业区建设，王府井百货等知名企业入驻我市。加快金融服务业发展。规范完善五大投融资公司，市商业银行与港中旅集团实现战略合作。引进银行5家，组建村镇银行4家。上市企业8家，居全省第三位。

（三）全面深化改革开放，发展动力活力持续增强。深化财政体制改革，“四权分离”新机制荣获第七届中国地方政府创新奖。深化教育体制改革，我市成为义务教育均衡发展等国家试点市。深化医药卫生体制综合改革，六县市全部成为国家县级公立医院改革试点。文化、土地、集体林权等改革顺利推进。2013年，实际利用境内外资金1107亿元，是2008年的4倍；进出口总额23亿美元，是2008年的1.5倍。

（四）统筹城乡一体发展，新型城镇化建设稳步推进。编制中心城市组团式发展规划，实施城市建设三年大提升行动，着力增强辐射带动能力。加快建设重大基础工程。举全市之力服务国家工程，南水北调总干渠全线贯通，配套工程顺利推进，以人为本、和谐征迁的做法受到习近平总书记肯定，创造了“顾全大局、无私奉献，情系群众、务实为民，开拓创新、奋发有为，众志成城、克难攻坚”的南水北调焦作精神，我市荣获全国南水北调工程征迁工作先进集体称号。谋划建设城市水系、郑焦城际铁路，新增国道276公里，新改建干线公路458公里。完善提升城市功能。更新公交车347台，新增绿地面积39万平方米，新改建一批城市道路、公园游园，供水、供暖、供气覆盖率进一步提高。加快棚户区改造，启动城中村改造，建成保障性住房4万余套。完成76条背街小巷整治。数字城管系统基本建成，智能交通管理系统覆盖面持续扩大。积极创建国家森林城市，森林覆盖率达27%。稳步推进新农村建设。新改建农村公路1572公里。建成新型社区住房近4万套。搬迁10个贫困村，6万人实现脱贫。解决了83万人饮水安全问题。

（五）着力保障改善民生，社会大局和谐稳定。持续增加民生投入，财政民生支出累计达497亿元，占财政支出69%。教育事业加快发展。提高义务教育生均公用经费标准，全面完成学前教育三年行动计划，累计完成1124个农村中小学校舍维修改造项目，我市荣获全国“两基”工作先进单位、全国推进义务教育均衡发展工作先进市称号，在全省率先实现职教强县全覆盖。卫生事业快速发展。提高城乡居民医保标准，全面实施基本药物制度，加快推进基层医疗机构标准化建设，基本公共卫生服务均等化水平不断提高。文体广电事业不断进步。市科技馆、图书馆建成并免费开放。实现了县县有图书馆、文化馆，乡乡有文化站，村村通广播电视、有农家书屋。行政村和社区体育健身工程实现全覆盖。人口计生工作稳步推进。出生人口性别比趋于平衡，我市成为全国创建幸福家庭活动试点。就业工作持续加强。大力推进就业创业，在全省率先实现人社服务站行政村全覆盖，城镇新增就业44万余人，农村劳动力转移就业25万余人。社会保障更加有力。城镇医保、新农合、城乡养老保险制度实现全覆盖，企业退休人员养老金连年增加，城乡低保、农村五保标准持续提高，住房公积金覆盖范围持续扩大。残疾人保障服务水平不断提升。实施敬老补贴制度，补贴标准全国领先。食品安全监管有效加强。在全国率先构建市县乡村四级监管网络，建立推行食品安全监管十项制度，荣获全国食品安全制度创新最佳事例奖。社会治理体制持续创新。城乡基层自治组织服务功能进一步增强，平安焦作建设扎实推进，应急管理体系不断完善，信访形势总体平稳，安全生产形势持续稳定好转。双拥共建深入开展。积极推进军民融合式发展，驻焦部队全面建设成效明显，支持地方建设作出重要贡献，我市成功实现全国双拥模范城四连冠、省级双拥模范城六连冠。

（六）持续转变政府职能，自身建设不断加强。深入推进依法行政，扎实创建法治型政府，自觉接受人大法律监督、工作监督和政协民主监督。深入开展“人民在心中、转型我先行”主题实践活动，扎实开展治庸、治懒、治乱、治散、治低“五治”行动，取消、下放和调整行政审批208项，政风行风不断好转。深化岗位权力风险防控，组建市公共资源交易中心，实施政府性投资建设项目“十个百分之百”管理，累计审减项目资金30亿元。认真落实中央八项规定和省市意见，“三公”经费明显下降，清理腾退办公用房近7万平方米。对口援疆工作扎实推进，统计调查、工商、民族宗教、检验检疫、气象、地震、档案、史志、对台、红十字会等工作都取得了新的成绩。

各位代表，2013年是全面贯彻落实党的十八大精神的第一年。一年来，我们坚持凝心聚力、转型攻坚、争创一流、绿色发展，主动转变思维方式、行为方式和发展方式，大力实施工业强市、科技强市，经济转型示范市和美丽焦作建设全面加快。

这是十大建设快速推进、成效明显的一年。集中要素资源，全力加快建设，城市水系引黄入焦干渠全线贯通，龙源湖水循环系统全面完工，灵泉湖、大沙河防洪生态治理等快速推进；郑焦城际铁路施工线路全部交付，跨黄河大桥工程完工；南太行生态景观带和林邓线绿化完成3600亩；国际太极拳文化交流中心暨全民健身中心主体结构建成；武西高速桃花峪黄河大桥建成通车；焦作东区热电机组建成投运；智慧旅游实现云台山景区网络全覆盖；职业技术学校新校区建成投用；焦作海关开关运行。

这是科技强市创新机制、加大投入的一年。突出科技对转型发展的支撑引领作用，压缩三公经费1000万元用于科技创新，年度科技三项费用达3650万元，居全省第3位。在全省首家开展企业科技创新能力评价，在全省首家试行创新券制度、科技贷款贴息制度，发放创新券5670万元、贷款贴息1852万元。与中关村国家自主创新示范区实现战略合作，中关村科技成果产业化基地落户焦作。我市成为国家知识产权试点城市，连续六次荣获全国科技进步先进市。

这是转型发展全面推进、步伐加快的一年。强力推进中国兵装焦作装备产业园等288个重点工业项目建设，多氟多电子级氢氟酸等项目竣工投产，全市规模以上工业企业达1175家，超亿元企业达691家。夏粮单产保持全省第一，农业产业化集群快速发展，农村土地流转率高于全国全省平均水平。《河南省云台山景区保护条例》颁布实施，我市成功创建省旅游标准化示范市。焦作电子商务产业园获批。澳洲联邦银行温县村镇银行正式开业，中证期货开业运营。新增直接融资157亿元，增长20%。

这是示范区建设上档升级、加快发展的一年。综合施策、全力支持，定期现场办公，在简政放权、科技强区、资源配置、项目建设等方面加大政策扶持力度。新规划49平方公里示范区产业集聚区，一期12平方公里基础设施建设全面启动。示范区成功创建国家新型工业化产业示范基地，是2013年度全省唯一一家。

这是经营城市力度加大、初见成效的一年。注重规划科学性，加强土地管理，重大事项一律集体研究、科学决策、严肃执行。严格公共资源管理，千方百计启动经营城市公共资源。加快推进南太行绕城高速、东海大道等大外环规划建设，城市框架不断拉大。强力开展城区违法建设、违法用地“双违”整治，拆除违法建筑140万平方米，清理违法用地2800亩。我市荣获全国国土资源节约集约模范市。

这是民生保障质量提高、水平提升的一年。财政用于民生支出133亿元，增支15亿元，增长13%。新建改建幼儿园103所，教育信息化水平居全省第2位。提高城乡基本公共卫生服务经费人均补助标准。新购置公交车100台，历年来数量最多。广泛开展舞台艺术送农民等群众文化活动。建成体育健身工程610个。在全省率先实行城镇职工和居民大病医疗补助。在全省率先推行农民工工资卡制度。我市成为省创建创业型城市试点。城乡居民社会养老保险参保率达99.6%，居全省第2位。

各位代表！过去的五年，是我市转型升级全面推进的五年，是经济社会快速发展的五年，是人民群众得到更多实惠的五年。这是省委、省政府和市委坚强领导的结果，是市人大、市政协、焦作军分区大力支持的结果，是全市人民共同奋斗的结果。在此，我代表市人民政府，表示衷心的感谢！

在肯定成绩的同时，我们也清醒地认识到，我市经济社会发展仍然存在不少矛盾和问题。一是思想观念相对滞后。发展理念不新，市场意识、经营观念不强，不适应新形势新要求。政府这只手用得多，市场这只手用得少，注重投入，不注重经营，比较效益不高。二是结构性矛盾依然突出。第三产业发展滞后，工业品集中于产业链前端和价值链低端的状况尚未扭转，传统优势产业支撑力下降，新的经济增长点还没有形成规模，自主创新体系尚不健全，发展的质量和效益还不高。三是承接产业转移能力仍然不强。基础设施不完善，资金保障、环境容量、土地供应等要素制约日益凸显，城市发展空间需进一步拓展，示范区、产业集聚区建设需进一步加大力度。四是民生工作与群众期盼还有差距。城乡低收入家庭生活还很困难，保障性住房建设、棚户区和城中村改造任务还很重，城市集中供暖覆盖率较低，城乡垃圾处理水平不高，教育、医疗等公共服务不足，交通拥堵现象日益突出。五是生态环境保护压力不断加大。大气污染、水污染尚未有效遏制，农村面源污染、土壤污染急需治理，雾霾问题日趋严重。六是政府作风转变还不到位。行政审批繁琐，效能效率不高，落实不力，推诿扯皮，中梗阻问题突出，用权不公、为政不廉等问题仍不同程度存在，影响了转型发展、加快发展步伐。对这些问题，我们首先从政府自身改起，采取有力措施，切实加快解决，让人民群众见到实实在在的成效。

二、今后五年目标任务

今后五年，是我市爬坡过坎、转型攻坚的关键时期，也是实现新跨越、再创新辉煌的重要战略机遇期。我国全面深化改革，我省全面实施中原经济区等三大国家战略，我市加快建设经济转型示范市，将释放新的动力、激发新的活力，为焦作跨越发展提供新的机遇。我市作为新型工业城市，装备制造、汽车及零部件等高成长性产业链条不断完善，生物医药、新材料等战略性新兴产业不断壮大，拥有一批占领行业制高点的科技创新型企业，拥有一批实干创新的领军型企业家，为我们加快新型工业化发展奠定了坚实基础；我市作为全国粮食高产区，种业科技全国领先，农业基础设施不断完善，农业集约化、专业化、组织化、社会化水平不断提升，为我们加快现代农业发展奠定了坚实基础；我市作为中国优秀旅游城市，太极文化博大精深，山水风光独具魅力，“四大怀药”功效独特，焦作服务享誉中外，为我们以拳以药为媒、点山点水成金，加快建设国际知名旅游城市奠定了坚实基础；我市作为对外开放优秀城市，海关及综合保税区功能不断完善，示范区、产业集聚区建设不断加快，城市水系等重大基础工程建设快速推进，为我们承接产业转移、发展开放型经济奠定了坚实基础；我市作为中原经济区核心区城市，积极推动与郑州交通融合，桃花峪黄河大桥建成通车，郑焦城际铁路年底通车，迎宾路南延及焦作黄河大桥等工程加快运作，为我们不断拉近与郑州的距离、促进焦郑融合发展奠定了坚实基础；焦作煤矿工人“特别能战斗”的光荣传统薪火传承，吃苦耐劳、诚信经营、精打细算、团结合作的怀商文化代代相传。这些都是我们攻坚克难、转型发展的强大正能量。

今后五年政府工作的总体要求是：深入贯彻落实党的十八大和十八届二中、三中全会精神，把改革开放贯穿于经济社会发展各领域各环节，按照“凝心聚力、转型攻坚、争创一流、绿色发展”总要求，坚持“一个战略、四个重点、十大建设”，更加注重强化创新驱动，更加注重优化经济结构，更加注重提高质量效益，更加注重保障改善民生，全面加快经济转型示范市和美丽焦作建设，为全面建成小康社会奠定坚实基础。

今后五年的主要目标是：

打造更具实力的富强焦作。全面加快经济转型示范市建设，大力实施工业强市、科技强市，以创新支撑引领转型升级，加快发展高成长性产业和战略性新兴产业，改造提升传统支柱产业。科学推进新型城镇化，推动城市南延发展，促进焦郑融合发展。到2018年，经济转型示范市建设取得显著成效，结构调整实现重大突破，自主创新体系更加完善，发展质量效益明显提高，人均主要经济指标高于全省平均水平，综合实力位居中原经济区前列，成为中原城市群重要增长极。

打造务实守信的文明焦作。广泛开展“争做美丽焦作人”活动，不断提高公民道德素质。深化行政体制改革，切实转变工作作风，彻底解决中梗阻问题，提升行政服务效能效率。加强信用体系建设，以政务诚信带动社会诚信。文明创建与经济建设同行，建立健全科学的考核评价体系，提速增量和提质增效并重，追求实实在在的发展。到2018年，公民文明素质和社会文明程度显著提高，力争成功创建全国文明城市。

打造和谐幸福的平安焦作。加强普法宣传教育，增强全民法律意识。加快发展社会事业，提升社会保障水平。创新社会治理方式，健全公共安全体系，促进公平正义，确保和谐稳定。到2018年，社会治理体制更加完善，基本公共服务更加均等，覆盖全民的社会保障体系基本建成，人民群众安居乐业，幸福感大幅提升。

打造生态宜居的美丽焦作。坚持绿色发展，加强生态环境保护，加快生态系统建设，全面建成城市水系，成功创建国家森林城市，成功创建全国生态文明先行示范区。到2018年，焦作青山相拥、绿水环绕，城在林中、景在城中，人居环境更优美，人与自然更和谐。

各位代表，只要我们全市上下勇于解放思想，大胆改革创新，凝心聚力、务实重干，以踏石留印、抓铁有痕的劲头抓重点、抓落实，焦作必将实现新的腾飞、再创新的辉煌！

三、2014年重点工作

今年经济社会发展的主要预期目标是：地区生产总值增长10%以上；公共财政预算收入增长11%；全社会固定资产投资增长22%；社会消费品零售总额增长14%以上；外贸出口增长10%；城镇居民人均可支配收入增长10%，农民人均纯收入增长10%；城镇新增就业6万人以上；人口自然增长率控制在6.5％。以内；节能减排完成省下达任务。

重点做好九个方面工作：

（一）着力深化改革开放。民之所望是我们施政所向。坚持从群众最期盼的领域、当前最急迫的事项、制约发展最突出的问题做起，力争一批重要领域和关键环节改革成为国家、省试点。在深化行政审批制度改革方面实现新突破。大刀阔斧简政放权，全面清理行政审批和行政服务类事项，该取消的一律取消，确需保留的，6月30日前实行权力清单制度，一律向社会公开，清单之外的，一律不得实施审批；大力改革项目审批制度，实行并联审批，自7月1日起，市本级行政审批时限由300个工作日压缩到40个工作日，创造焦作效率，走在全省前列。在加强财源建设方面实现新突破。把财源建设摆上重中之重位置，多措并举，以效益为导向，改革支出方式，优化支出结构，重点支持转型升级项目，重点支持科技创新型企业加快发展，培植新的经济增长点、培植新的财源，形成经济总量增加、产业结构优化、财政收入增速质量提高的良性循环，确保财政收入增速和质量在全省晋位升级。深化财税体制改革，实施全面规范、公开透明的预算制度，建立跨年度预算平衡机制，建立权责发生制的政府综合财务报告制度。开展综合治税，规范税费征管。高度重视政府债务管理，有效防范和化解债务风险。在深化教育卫生改革方面实现新突破。加快建设义务教育均衡发展等国家试点市，积极推进职教攻坚二期工程，大力发展民办教育。深化县级公立医院综合改革，推进医保支付方式、全科医生执业方式和服务模式改革，扩大基本药物制度实施范围，启动医疗联合体建设，探索实施保险机构参与的新农合大病保险制度，大幅提升医疗保障水平，为群众提供更加优质的医疗服务。在深化农村改革方面实现新突破。坚持最严格的耕地保护制度，慎重稳妥推进农村土地制度改革，先行先试、探索建立城乡统一的建设用地市场，全面完成农村宅基地、集体建设用地使用权确权登记发证工作，探索宅基地有偿退出机制，积极争取开展宅基地制度改革试点，研究完善农民资产到资本、资金转化的推进路径，显化农民房产权、财产权。同时，加快推进文化、金融、国企、投融资体制等各项改革。

坚持把开放招商作为“一举应多变、一招求多效、一优带百通”的综合性战略举措，大力开放招商。全面拓宽开放领域。落实统一的市场准入制度，开放一切可以放开的领域，鼓励境内外投资者进入旅游、金融、商务、物流等服务业，鼓励境内外资金进入城建、农业、教育、卫生等领域。强力推进开放招商。更加注重科技招商、以商招商、集群招商，坚持重落地、重投产、重带动，引进更多好项目、大项目。大力发展楼宇经济，积极争取跨国公司和大型企业在我市设立地区总部。积极发展开放型经济。做大做强省级汽车零部件出口基地，创建省级精细化工出口基地，新增海关双A类企业2家。发挥焦作海关优势，加强与青岛港、连云港等港口合作，吸引豫西北、晋东南企业办理业务，打造外贸综合服务区域中心城市。持续优化开放环境。外来企业、本地企业都是重要的经济增长点，都是加快发展的主力军。深化开放提升年活动，对外来企业、本地企业一视同仁，对投资项目一律落实优惠政策，一律提供保姆式、零距离服务，让企业高高兴兴投资、顺顺利利办事、踏踏实实发展。

（二）着力推进十大建设。今年安排市重点项目（第一批）319个，总投资2070亿元，年度计划投资528亿元。坚持把十大建设作为打基础、增后劲、管长远的重大工程强力推进。城市水系建设方面。实施新河治理工程，实施群英河等4个城市河道整治、景观水利用工程，完成影视湖、龙寺、圆融水库主体工程，完成大沙河城区段防洪治理工程，建成带状湿地公园。铁路建设方面。加快站南广场征迁建设，郑焦城际铁路建成通车。推进铁路货运北站搬迁前期工作。生态建设方面。完成高速公路两侧绿化提升工程，完成龙寺公园等地质环境综合治理项目，推进北部浅山区生态治理恢复，打造带状公园景观。海关及保税区建设方面。加快综合保税区建设，开工建设通港物流园区三期。示范区和产业集聚区项目建设方面。新开工10亿元以上项目24个，年度完成投资120亿元以上。信息化建设方面。加快财税信息共享平台、教育信息化和智慧医疗建设，深入推进电子政务公共平台、智能交通综合信息平台建设。国际太极拳文化交流中心暨全民健身中心建设方面。保质量、保进度，建成优质工程、精品工程。能源建设方面。龙源电厂、焦作电厂异地扩建工程各试运行一台机组，启动丰收路以南、焦郑高速以北城区电网归并改造，全面完成城区居民小区无人承接配电设施改造任务。公路建设方面。加快武云高速建设，启动云台山到省界段高速前期工作，力争南太行绕城高速开工建设。职教园区建设方面。加快推进技师学院新校区项目建设。

（三）着力加快工业转型。坚持工业强市，大力开展工业转型升级攻坚年活动，壮大工业整体实力。强力推进重点产业优化升级。实施“330”重点企业培育工程，推进100个超亿元转型升级重点项目建设，力争完成投资238亿元，41个项目竣工投产。把战略性新兴产业作为转型升级重点，聚焦政策、聚集资源、聚合力量，强力推进智能物联机器人、卓林数码电脑零部件、金宇阳电容触摸屏、华晶大单晶金刚石、超威电源混合动力汽车电池、辅仁药业小容量注射剂等项目，加快推动电子信息、新材料、新能源、节能环保、生物医药等产业向集群化规模化发展，尽快做大做强，培植新的增长板块。突出三大国家级产业基地建设，坚定不移推动传统支柱产业改造升级，推动产业链向末端延伸、价值链向高端进军。加快建设大江重工中高端液压支架、科瑞森成套设备等项目，全力打造国家先进装备制造基地；加快建设风神轮胎高性能乘用子午胎、中内配新型节能环保发动机气缸套等项目，发展壮大国家汽车零部件特色产业基地；加快建设佰利联氯化法钛白粉、多氟多含氟精细化学品等项目，全力打造全国现代循环化工产业示范基地。加快建设蒙牛乳业长效酸奶生产线、亿腾食品速冻及熟食工程等项目，做大做强食品产业。按照国务院进退并举部署，对接国家产业政策导向和市场走向，大力扶持有一定规模、科技含量高、市场前景好、盈利空间大的企业和项目，对产能过剩、技术落后、转型无望的企业不予支持。统筹推进城区和县域工业发展。加大城区工业支持力度，激发城区工业发展活力，培育一批骨干龙头企业，推动城区工业全面振兴，做强城区经济，提升辐射带动能力。加快县域经济发展，努力在全省争先进位。加快示范区和产业集聚区建设。坚定不移实施示范区科技强区战略，突出产城融合，加快项目建设，提高承载能力，增强综合实力，打造我市新的经济增长极，努力走在全省示范区前列。推动9个省级产业集聚区完善产业配套、壮大产业集群、加速提质发展，争创省“十强十快十先进”产业集聚区。强化企业服务。完善工业投融资平台，支持实体经济发展。完善市领导联系帮扶重点企业制度，实行首席企业服务员制度，健全服务企业长效机制。大力推进企业减负，对涉企部门继续电视问政，组织企业评价打分，对排名靠后的严肃问责。

（四）着力强化科技支撑。坚持科技强市，以创建国家创新型试点城市、建设国家知识产权试点城市为载体，大力构建具有焦作特色的自主创新体系。加大科技创新投入。市本级科技投入再翻一番，大力实施创新券、贷款贴息制度，重点扶持纳税百强、科技创新能力百强企业扩大优质产能。打造科技创新平台。不断深化与国家级自主创新示范区、科研机构、高等院校产学研合作，加快建设中关村科技成果产业化基地、中科院科技成果转移转化焦作分中心，推进国家煤炭安全生产技术创新中心建设，创建一批国家级研发平台、院士工作站。实施创新企业培育工程，培育一批国家高新技术企业。河南理工大学科技园一期建成投用，高新区力争成功创建国家级高新区。突出创新支撑引领作用。鼓励支持科技人员创新创业，大力引进科技领军人才、创新团队，大力引进新技术、新工艺、新成果，加快科技成果转化应用；实施重大科技专项、专利导航试点工程，突破一批核心技术，形成一批自主知识产权，增强企业核心竞争力。推动科技体制机制创新。完善科技强市常态化工作机制，健全企业科技创新能力评价服务机制，探索建立科技风险投资机制，实施专利抵押贷款制度，激发科技创新动力活力，依靠科技支撑、创新驱动打造焦作经济升级版。

（五）着力推进新型城镇化发展。用战略思维和长远眼光强化经营城市理念，积极稳妥、科学推进新型城镇化。加快农业转移人口市民化。以人的城镇化为核心，坚持产业为基、就业为本，强化住房、教育“两牵动”，促进城镇基本公共服务常住人口全覆盖。加快户籍制度改革，重点解决一批已进城就业定居农民工落户、一批城中村居民成建制转户，有序转移一批农村富余劳动力。推进城市组团式发展。加快焦修博武一体化，加快焦郑融合，完善中心城市组团式发展规划，积极推进城市组团、功能区和中心镇规划建设，积极推进示范区“十纵八横”主干路网建设，加快大外环规划建设，谋划推进迎宾路南延及焦作黄河大桥项目，促进交通链接、产业对接、生态共建、服务共享。提升城市承载能力。实施中原路延伸等工程，完成龙源路延伸、普济路大修等项目，再购置一批公交车，新增供水管网40公里、燃气管网80公里。突出抓好供热管网建设，开工建设新园路、工业路、塔南路供热管网，利用社会资本建设焦作东区热电机组、龙源电厂配套供热管网，新增供热面积100万平方米以上，让更多群众寒冬用上暖气、生活更加舒适。强化城市管理。大力推进城乡接合部环境卫生整治，加强背街小巷、建筑垃圾、户外广告、市政设施、市容秩序等专项整治，提高城市精细化管理水平。创新城市建设体制机制。探索发债等方式拓宽城市建设融资渠道，鼓励社会资本以特许经营等方式参与城市基础设施建设运营，力争促成一批重大项目。加快建设南水北调配套工程，探索采用城中村改造模式，推进南水北调工程两侧绿化带及基础设施建设。严格土地市场管理，加强土地收储，扩大国有建设用地公开出让范围，经营性建设用地一律公开招拍挂，坚决杜绝商住用地出让中“再协议”和“跑冒滴漏”；强力推进“双违”整治，严格属地管理、部门管理，确保“双违”存量减少、增量为零。

（六）着力发展现代农业。全面落实强农惠农富农政策，加快构建新型农业经营体系，强力推进新型农业现代化发展。提高粮食生产能力。推进百千万工程建设，新增高标准粮田25万亩，确保粮食稳产高产。构建新型农业经营体系。积极推进农业经营方式创新，加快农村土地流转，大力培育专业大户、家庭农场、农民合作社、农业企业等新型农业经营主体。健全农畜产品质量安全监管体系，实施农产品品牌战略，打造一批市场上叫得响的名牌农产品。加快农业产业化集群发展。进一步壮大蔬菜、食用菌、畜牧等特色农业，积极发展怀药养生等保健品产业，培育发展都市生态农业，18个农业产业化集群销售收入力争突破550亿元。提升农机保障水平。新增农机具5000台，主要粮食生产机械化水平保持在96%以上。加强农业基础设施建设。推进孤山湖水库项目，加快中小河流治理、病险水库加固工程，发展节水灌溉面积6万亩，治理水土流失面积40平方公里。今年再解决15万农村群众饮水安全问题。强化农业科技服务。加强农业科技创新，推进种业联合重组，规范种子市场，主要作物良种覆盖率达到98%以上。加快农技推广区域站建设，提高农业科技服务水平。加强气象现代化建设，进一步完善气象防灾减灾体系。推进新农村建设。科学编制实施新农村规划，加快建设基础设施和公共服务设施，新建农村公路200公里。开展农村环境卫生集中整治行动，加大投入，推进村庄净化、硬化、绿化、亮化、美化，建设“美丽乡村”。

（七）着力提升服务业发展水平。认真落实支持服务业发展优惠政策，大力发展现代服务业，推动服务业发展提速、比重提高。加快发展文化旅游业。围绕打造国际知名旅游城市，强力推进文化旅游园区和重大项目建设，年度完成投资30亿元。编制完成黄河生态文化旅游带等专项规划，谋划实施沿黄旅游大道、南太行旅游快速通道延伸工程。规划建设中心城区旅游餐饮、购物、娱乐街区，大力开发旅游商品，完善吃住行游购娱产业链条，拓展国内外客源市场。加快发展太极拳文化产业，推动太极拳申遗，完成云台山旅游股份有限公司上市申报。推进旅游信息化、标准化建设，完善旅游公共服务体系，创建国家级旅游服务综合标准化示范城市。加快发展金融服务业。积极推进台湾第一银行在修武、博爱设立村镇银行，引导金融机构在产业集聚区、旅游景区设立分支机构。拓宽保险服务领域，不断扩大保险覆盖面。加快发展资产管理、专业保险等新兴金融业。完善财政性资金存放商业银行评价激励机制，提高金融机构信贷投放积极性。探索中小微企业贷款风险补偿，努力破解中小微企业融资难、融资贵问题。加快推进企业进入多层次资本市场，做好上市（挂牌）后备企业指导服务，力争一批企业成功上市（挂牌）。加快发展商贸物流业。大力推动商贸企业建设网上商城、中小微企业开设网店，大力支持电商服务平台建设，争创省级电子商务产业示范园区；加快推进物流园区、现代物流公共信息平台建设，大力发展电商速递等专业物流。加快发展商务中心区和特色商业区，开工建设动漫产业园。全面启动新河商务中心区项目，高标准、高水平规划建设，打造焦作CBD，打造三产发展新板块、新亮点。

（八）着力加强生态文明建设。把生态文明建设融入经济社会发展全过程，加快建设美丽焦作。实施蓝天工程。全面完成火电、水泥行业烟气除尘、脱硫脱硝治理，全部关停城区集中供热供气区燃煤锅炉，加快实施西部工业产业集聚区等重点区域大气环境综合整治，提前完成造纸等21个行业落后产能淘汰任务，强力推进扬尘污染控制、煤场专项整治、清洁能源推广等工作，努力使雾霾天气减少、空气质量好转。实施清水工程。所有污水处理厂完成提标改造，城区供水管网覆盖区自备井一律关闭，城区主干污水管网全部建成，城市生活污水处理率达到85%以上；大力开展河流专项治理，确保省控断面排放达标。实施乡村清洁工程。加快农村污水垃圾处理设施建设，加强农村面源污染治理和土壤污染防治，强力推进重金属污染治理。加强资源节约和综合利用。大力实施工业、建筑等八大节能工程，做好资源枯竭城市转型年度绩效评价工作，深入推进国家资源综合利用示范基地建设。提高主要污染物排污费标准，积极推进排污权交易试点，完善生态补偿机制。加强生态体系建设。深入创建国家森林城市，加快太行山绿化、平原绿化、生态廊道网络和黄河滩防护林体系建设，重点做好南太行生态景观带、农田林网和村镇绿化，积极推进城市道路高标准绿化示范工程，完成“七河八路十二园”绿化工作，完成森林资源培育18.4万亩，努力构建“一区两带三网多点”林业生态网络。我们必须始终坚持绿色发展，坚决向污染宣战，对排放不达标企业、偷排滥排企业一律关停，决不上污染项目，决不要黑色、灰色、雾霾GDP，还老百姓蓝天碧水，呵护我们的共同家园。

（九）着力保障改善民生。继续加大民生投入，扎实办好省市十项重点民生工程，进一步提升基本公共服务水平，努力让老百姓过上好生活。加快发展社会事业。在新城区开工建设丰泽园小学、映湖路小学、韩公教育园区小学幼儿园，对老城区教育资源进行优化配置，完成100个农村中小学标准化项目，加快建设示范性综合实践基地。大力推进焦大、师专升本工作，促进高等教育发展。优化卫生资源布局，重点推进市二医院新城区医院、妇幼保健院儿童医院综合病房楼项目建设，完成市人民医院全科医师培训基地、四医院综合病房楼建设。继续实施舞台艺术送农民送基层等文化惠民工程，做好博物馆新馆、群艺馆等项目前期工作。办好省十二届运动会暨首届全民健身大会，创建省首批全民健身示范市。落实国家生育政策，提高出生人口素质，加快省人口均衡型社会综合配套改革示范市建设。加强就业创业工作。深入推进统筹城乡就业创业试点市、创业型城市建设，做好高校毕业生、农村转移劳动力、城镇困难人员等重点群体就业工作，鼓励事业单位人员离岗创业，城镇登记失业率控制在4.5%以内。加大社会救助力度。尽最大努力保障城乡低保、困难群体基本生活，今年强力推进社会救助制度改革，安排2000万元提高城乡低保补助水平；安排1000万元建立城乡医疗救助对象医疗费用补充保险，符合规定的医疗费用在现有基础上报销到95%；安排1000万元加强临时救助，妥善解决城乡困难群众突发性、临时性生活困难。新增财力足额保障，履行好政府兜底责任。强化社会保障。企业退休人员基本养老保险金水平提高10%，对城镇职工和居民医疗费用实行按比例上不封顶报销。积极推动城镇职工养老保险与城乡居民养老保险衔接，城镇职工医保与居民医保、新农合衔接。进一步扩大住房公积金覆盖面。完善残疾人社会保障和服务体系。加快推进棚户区改造，推动城中村改造实现新突破，新开工保障性住房7925套、建成8282套。大力支持社会力量兴办养老机构，推进社会养老服务体系建设，启动市福利院老年公寓楼项目，新建农村幸福院200家，新增养老床位3000张以上。积极创新社会治理。深入开展“争做美丽焦作人”活动，大力推进和谐社区建设，积极推进文明城市创建。建设平安焦作，深入开展普法教育，加强法律援助工作。完善信访工作制度，及时就地化解矛盾。加强安全生产监管，坚决遏制重特大事故发生。加强应急能力建设，提升应急管理水平。依法规范宗教场所建设。大力发展志愿者事业。深化双拥创建成果，巩固军政军民团结，促进军民融合深度发展。全面推进食品药品监管体制改革，开工建设食品药品检测中心，健全完善监管体系，切实保障食品药品安全。严厉打击和处置非法集资，确保社会大局和谐稳定。

四、以改革创新精神打造务实重干的人民政府

深入开展党的群众路线教育实践活动，突出为民务实清廉，加快转变工作作风，加快转变政府职能，努力建设服务型政府、法治政府、创新政府、廉洁政府。

（一）坚持改进作风，建设服务型政府。严格执行中央八项规定和省市意见，健全改进作风常态化制度，坚决遏制“四风”，提升政府执行力和公信力。喊破嗓子不如甩开膀子，大力倡导务实重干，把更多时间花在听民声、知民情、解民忧上，把更大功夫下在解决问题、跟踪督导、狠抓落实上，只做有用功、不做无用功、杜绝做负功。严格落实国务院约法三章，政府性楼堂馆所一律不新建改扩建，财政供养人员只减不增，“三公”经费只减不增，政府过紧日子，让老百姓过好日子。

（二）坚持依法行政，建设法治政府。自觉接受人大及其常委会的法律监督、工作监督，自觉接受人民政协的民主监督。严格按照法定权限和程序履行职责、行使权力，把政府工作全部纳入法治轨道。改革行政执法体制，规范行政执法行为。强化审计、监察等专门监督，主动接受人民群众监督和舆论监督，深入推进政务公开，让人民监督权力，让权力在阳光下运行。

（三）坚持转变职能，建设创新政府。处理好政府和市场的关系，使市场在资源配置中起决定性作用和更好发挥政府作用，强化政府公共服务、市场监管、社会管理、环境保护等职责，推动政府职能加快向创造良好发展环境、提供优质公共服务、维护社会公平正义转变。理顺权责关系，规范机构设置，推进政府机构改革和事业单位分类改革，推进政府向社会购买服务改革。创新思维方式，转变行为方式，强化市场理念、经营理念和亩产论英雄理念，注重成本最低化、收益最大化，更多运用市场这只手破解难题、推动工作、促进发展。

（四）坚持清廉从政，建设廉洁政府。严格落实党风廉政建设责任制，全面加强惩治和预防腐败体系建设，深化岗位权力风险防控，健全用制度管权、管事、管人的长效机制，保证政府权力规范运行。加强公共资源交易监管，强化纠风和专项治理，坚决查处损害群众利益行为，坚决查处腐败案件，严惩腐败分子，以廉政建设和反腐败斗争的新成效保障发展、取信于民。

各位代表！美好的前景鼓舞着我们，人民的期盼激励着我们！让我们在省委、省政府和市委的正确领导下，解放思想、开拓创新，务实重干、砥砺奋进，全面加快经济转型示范市和美丽焦作建设，谱写人民美好生活新篇章！
